# Avoiding UV light

**DOI:** 10.7554/eLife.92535

**Published:** 2023-10-18

**Authors:** Maria Sachkova, Vengamanaidu Modepalli

**Affiliations:** 1 School of Biological Sciences, University of Bristol Bristol United Kingdom; 2 Marine Biological Association of the UK Plymouth, Devon United Kingdom

**Keywords:** UV avoidance, nitric oxide, zooplankton, *Platynereis dumerilii*, neural circuits, *P. dumerilii*

## Abstract

The larvae of an annelid worm use nitric oxide signalling to activate the neural pathways needed to swim away from the harmful ultraviolet light of the sun.

**Related research article** Jokura K, Ueda N, Gühmann M, Yañez-Guerra LA, Słowiński P, Wedgwood KCA, Jékely G. 2023. Nitric oxide feedback to ciliary photoreceptor cells gates a UV avoidance circuit. *eLife* **12**:RP91258. doi: 10.7554/eLife.91258.

Living deep within our oceans, lakes, and ponds are small animals known as zooplankton which typically rise to the surface of the water at night and sink towards the bottom during the day. This synchronised movement helps zooplankton avoid harmful ultraviolet (UV) light and escape diurnal predators that hunt during the day (Malloy et al., 1997).

Most marine invertebrates progress through a ciliated larval stage during their life cycle, and this larva will swim freely like zooplankton before settling on the seafloor and transforming into an adult. During this free-swimming stage, the ciliated larvae also avoid UV light, making them a useful model for studying how zooplankton behave. In the larvae of the annelid worm *Platynereis dumerilii*, this response is controlled by ciliary photoreceptor cells which detect UV wavelengths via a light-sensitive protein known as c-opsin1 (Verasztó et al., 2018; Conzelmann et al., 2013; Arendt et al., 2004). The larvae of other marine invertebrates also use this mechanism to sense UV light (Jékely et al., 2008). However, it was unclear how this sensory input is relayed to the parts of the nervous system that trigger the larvae to swim downwards away from the sun. Now, in eLife, Gáspár Jékely and colleagues – including Kei Jokura as first author – report that *P. dumerilii* larvae use the gaseous signalling molecule nitric oxide to pass on this information (Jokura et al., 2023).

The team (who are based at the University of Exeter, University of Bristol, Okinawa Institute of Science and Technology and University of Heidelberg) found that the enzyme responsible for generating nitric oxide, nitric oxide synthase (or NOS for short), is expressed in interneurons that reside in the apical organ region, the part of the larva that receives sensory input. Previously collected electron microscopy data from the whole larval body of *P. dumerilii* was then analysed (Williams et al., 2017), which revealed that these NOS-expressing interneurons lay immediately downstream of UV-sensing ciliary photoreceptor cells.

To further test whether nitric oxide is involved in UV avoidance, Jokura et al. studied *P. dumerilii* larvae that had been genetically modified so that any nitric oxide produced by these animals emits a fluorescent signal. They found that UV exposure led to higher levels of fluorescence in the part of the larva where the NOS-expressing interneurons project their dendrites and axons. Furthermore, mutant larvae lacking the gene for NOS did not respond as well to UV light, an effect that has been observed previously in mutant larvae that do not have properly working c-opsin1 photoreceptors (Verasztó et al., 2018). These findings confirm the role of nitric oxide in UV-avoidance.

Next, Jokura et al. investigated how nitric oxide signalling affects the activity of ciliary photoreceptor cells using a fluorescent sensor that can detect changes in calcium levels: the more calcium is present, the more active the cell. UV light exposure caused the ciliary photoreceptors to experience two increases in calcium. This biphasic response depended on c-opsin1 and nitric oxide molecules being retrogradely sent from the NOS-expressing interneurons back to the ciliary photoreceptor cells.

Jokura et al. also identified two unconventional nitrate sensing guanylate cyclases (called NIT-GC1 and NIT-GC2) which mediate nitric oxide signalling in the ciliary photoreceptor cells. These proteins are located in different regions of the photoreceptor and may therefore be involved in different intracellular signalling pathways. Experiments with mutant larvae lacking NIT-GC1 confirmed that this protein is necessary for retrograde nitric oxide signalling to ciliary photoreceptor cells. This leads to a delayed and sustained activation of the ciliary photoreceptors, which then drives the circuit during the second increase in calcium. A mathematical model that analysed the dynamics of the neural circuit, and individual cells within it, confirmed that the magnitude of the nitric oxide signal depends on the intensity and duration of the UV stimulus.

In conclusion, Jokura et al. propose that when *P. dumerilii* larvae are exposed to UV light, this activates ciliary photoreceptors, which, in turn, triggers postsynaptic interneurons to produce nitric oxide (Figure 1). The nitric oxide signal is then sent back to the ciliary photoreceptors, causing them to sustain their activity (even once the stimulus is gone) via an unconventional guanylate cyclase. This late activation inhibits neurons which promote cilia movement. Jokura et al. propose that slowing the beat of certain cilia may rotate the larva so that its head is pointing downwards, causing it to swim away from UV light at the water surface.

**Figure 1.**
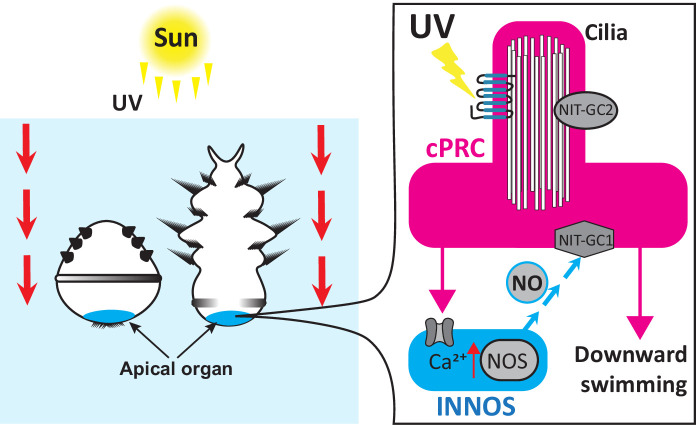
The neural circuit that instructs ciliated larvae to avoid UV light. Two- and three-day-old larvae of the annelid *Platynereis dumerilii* swim downwards to avoid UV exposure from the sun. The UV light is detected by ciliary photoreceptor cells (cPRCs, pink) which activate interneurons (INNOS, blue) downstream by increasing their calcium (Ca^2+^) levels. This triggers the enzyme nitrogen oxygen synthase (NOS) to generate the gaseous signalling molecule nitric oxide (NO) which is sent back to the ciliary photoreceptors. Nitric oxide interacts with a nitrate sensing guanylate cyclase (NIT-GC1) which sustains the activity of the ciliary photoreceptors. This signal activates a chain of downstream neurons resulting in the larvae swimming downwards away from UV light at the water surface.

As animals have evolved, their light-response systems have become increasingly sophisticated, especially with the addition of neurons which have further refined this process. Nitric oxide is an ancient signalling molecule that regulates many processes in animals, and its newly discovered role in the ciliated larvae of *P. dumerilii* may help researchers find missing connections in the light-sensing pathways of other marine invertebrates.
